# Type of e-liquid vaped, poly-nicotine use and nicotine dependence symptoms in young adult e-cigarette users: a descriptive study

**DOI:** 10.1186/s12889-020-09056-y

**Published:** 2020-06-12

**Authors:** Erika N. Dugas, Marie-Pierre Sylvestre, Jennifer O’Loughlin

**Affiliations:** 1 Centre de recherche du centre hospitalier de l’Université de Montréal (CRCHUM), 850 Saint-Denis (S03-468), Montreal, Quebec H2X 0A9 Canada; 2 Department of Social and Preventive Medicine, Université de Montréal, Montreal, QC Canada

**Keywords:** Young adults, E-cigarettes, Cigarettes, Nicotine, Nicotine dependence, Cannabis

## Abstract

**Background:**

Data are lacking on type of e-liquid vaped among e-cigarette users. Further, few studies assess all sources of nicotine used by e-cigarette users to assess whether poly-nicotine use relates to nicotine dependence (ND). The objectives were to describe young adult e-cigarette users by: (i) type of e-liquid vaped; (ii) poly-nicotine use; (iii) ND symptoms; and (iv) attempts to quit conventional cigarettes.

**Methods:**

Data were available in cycle 23 of a longitudinal investigation on the natural course of cigarette smoking and ND. A total of 775 young adults (44% male; mean (SD) age 30.5(1.0)) completed mailed self-report questionnaires in 2017–20.

**Results:**

Of 775 participants, 149 (19.2%) reported past-year e-cigarette use. Overall, 55.0% of e-cigarette users had used cannabis-containing e-liquid (31.5% vaped cannabis e-liquid exclusively); 50.4% used nicotine-containing e-liquid (23.5% vaped nicotine e-liquid exclusively); and 39.9% used e-liquid without nicotine (8.7% vaped e-liquid without nicotine exclusively). Most e-cigarette users (82.6%) used other nicotine-containing products including conventional cigarettes (72.5%); 60.8% reported ND symptoms, rising to 79.4% among those who vaped nicotine-containing e-liquid. Finally, 29.0% tried to quit conventional cigarettes using e-cigarettes in the past-year, but only 16.7% found them helpful.

**Conclusions:**

E-cigarettes now appear to appeal to a broader market than smokers who want to quit. More than half of young adult e-cigarette users vaped cannabis-containing e-liquid in the past year while only one-quarter had used e-cigarettes to assist with cessation. Most e-cigarette users used multiple nicotine-containing substances (including combustible cigarettes) which were associated with reports of ND symptoms.

## Background

E-cigarette use is common in Canada. In 2017, 23–29% of persons age 15–34 years reported ever trying e-cigarettes and 6% reported daily use [1]. Between 2017 and 2018, a dramatic 74% increase in past-month use was observed among 16–19-year-old Canadians [2]. In the same time period, current e-cigarette use increased 46% in 18–24-year-old Americans [3]. Because e-cigarette use is escalating among adolescents and young adults [1, 2], there is growing concern that the tobacco industry is targeting young people [4, 5] with e-cigarette marketing strategies that appeal to this age group including colorful packaging, a significant online presence [4–6], celebrity endorsements [5] and more than 7000 flavors, most of which are fruit- or confectionary-flavored [7]. New e-cigarette products targeting young people appear on the market with regularity. For example, e-cigarettes designed specifically to vape cannabis have become increasingly popular [8–11] and are associated with lower perceived risk [11] and increased use [12]. Although there is an extensive literature on e-cigarettes and smoking cessation, the evidence that e-cigarettes with or without nicotine help smokers quit remains inconsistent in systematic reviews [13–16] and randomized controlled trials [17–20]. Additional concerns include the presence of nicotine in e-cigarettes which could hinder cessation efforts and delay cessation [4], the impact of e-cigarette use on re-normalizing smoking [4], smoking re-uptake in former smokers [4], and growing evidence that e-cigarettes might be a gateway to cigarette smoking initiation in youth [4, 21–23]. We add poly-nicotine use (i.e., use of multiple nicotine-containing substances concurrently) to this list. Poly-nicotine use is common in young people [1, 4, 24, 25]; it is estimated that only 1.4% of U.S. adults are sole e-cigarette users [26].

More recently, there has been widespread alarm about vaping-induced respiratory injuries in the US [27] and Canada [28]. A US Center for Disease Control (CDC) special announcement in October 2019 [29] reported that most lung injury cases involved tetrahydrocannabinol (THC)-containing products and its recommendations included, among others, that: people should not vape products containing THC or modify e-liquid; using any type of e-cigarette is unsafe; nicotine is highly addictive and can harm the developing brain; and e-cigarettes should not be used by youth or young adults. In light of this report, it is critical to better describe young e-cigarette users and in particular, whether e-liquid used contain cannabis. In addition, few studies assess all sources of nicotine used by e-cigarette users and assess whether poly-nicotine use relates to nicotine dependence. The objectives of this study were to describe young adult e-cigarette users by: (i) type of e-liquid vaped in the past year (i.e., with nicotine, without nicotine, with cannabis); (ii) poly-nicotine use (i.e., concurrent use of multiple nicotine-containing substances); (iii) nicotine dependence (ND) symptoms; and (iv) attempts to quit conventional cigarettes using e-cigarettes.

## Methods

Data were drawn from the Nicotine Dependence in Teens (NDIT) study, a longitudinal investigation on the natural course of nicotine dependence of 1294 students recruited in 1999–2000 from all grade 7 classes in a purposive sample of ten Montreal-area high schools [30]. NDIT used a school-based sampling strategy to recruit participants. High schools (*n* = 13) in or near Montreal were selected in consultation with local school boards and school principals to include a mix of: (i) French- and English-language schools; (ii) urban, suburban and rural schools; and (iii) schools located in high, moderate and low socioeconomic status neighborhoods. Private schools were excluded. Two schools were excluded because of a low return of parental consent forms, and one school was excluded because school administrators could not guarantee continued participation in NDIT after the first year of the study. The total number of schools retained was 10. All grade 7 students (mean age 12.8 years) in the study schools including special needs students, were invited to take part of the study. Data were collected in self-report questionnaires administered at school every 3–4 months from grade 7 to 11, and in three cycles post-high school when participants were age 20.4, 24.0 and 30.5 years on average. The current analysis used data collected in 2017–20 when participants were age 30.5 years.

The study was approved by the Ethics Research Committee of the Centre de Recherche du Centre Hospitalier de l’Université de Montréal. Participants provided written informed consent.

### Study variables

*E-cigarette liquid -* Participants were asked how often (never; less than once a month; 1–3 times per month; 1–6 times per week; every day) in the past 12 months they had used e-cigarettes with nicotine; without nicotine; or to vape marijuana, hash oil, liquid or wax. For several analyses, responses were recoded as no (never)/yes (all other responses).

*Past-year cigarette smokers –* Ever smokers were asked to self-identify into one of the following: I have smoked cigarettes, but not at all in the past 12 months; I smoked cigarettes once or a couple of times in the past 12 months; I smoke cigarettes once or a couple of times each month; I smoke cigarettes once or a couple of times each week; I smoke cigarettes every day. Responses were recoded no (I have smoked cigarettes, but not at all in the past 12 months)/yes (all other responses).

*Current smoking* was measured by: “During (last month), on how many days did you smoke cigarettes, even just a puff?” Participants were categorized as current smokers (i.e., smoked in the past month) if they responded smoking on one or more days in the past month.

*Other tobacco products* - Participants were asked how often in the past 12 months they had used: (i) flavored cigarettes or cigarillos; (ii) cigars, pipe, bidis, chewing tobacco and/or snuff; (iii) cigarillos; and (iv) waterpipe. Because of low frequency of use, all products were combined into a single variable called “*other tobacco products”*. Responses were coded yes (i.e., used one or more)/no (i.e., did not use any).

*Cannabis mixed with tobacco –* Participants were asked how often (never; less than once a month; 1–3 times per month; 1–6 times per week; every day) in the past 12 months they used marijuana, cannabis or hashish mixed with tobacco?" Responses were recoded no (never)/yes (all other responses).

*Number of other nicotine-containing substances* used in the past year was the sum of positive responses for conventional cigarettes, other tobacco products and cannabis mixed with tobacco (range 0–3) (excluding e-cigarettes with nicotine).

There is evidence that former smokers continue to experience ND symptoms short- and long-term [31, 32]. Further, young never smokers who live in environments with exposure to second- and third-hand smoke report ND symptoms [33, 34]. In this study, questions on perceived ND and cravings were measured among all ever and non-smokers (including never smokers and quitters), whereas withdrawal symptoms was measured in past 3-month smokers only. *Perceived (*cigarette-related) *ND* was measured by: “Even if you do not currently smoke cigarettes, how addicted to smoking cigarettes are you …? ” (i) physically; (ii) mentally. Responses (not at all; a little bit; quite; very) were recoded (yes/no) according to whether participants reported any positive response.

*Craving* was measured by: “Even if you do not currently smoke cigarettes, how often do you … crave a cigarette?” For analysis, response choices were recoded yes (rarely, sometimes, often)/no (never).

*Withdrawal symptoms* was measured among ever smokers only by: “When you cut down or stop using cigarettes, or when you are not able to smoke for a long period (like most of the day), how often did you experience … feeling a strong urge or need to smoke”. Responses were recoded yes (rarely, sometimes, often)/no (never). Convergent construct validity of the craving and withdrawal indicators were demonstrated previously against quit attempts and smoking status [35].

Use of *e-cigarettes as a cessation aid* was measured among ever smokers only by: (i) “Did you ever try any of the following to help you quit smoking cigarettes...” (a) electronic cigarettes with nicotine (yes/no), (b) electronic cigarettes without nicotine. (yes/no); (ii) “If yes, was this in the past 12 months?” (yes, no); and (iii) “Did it help you to quit?” (yes, no). Use of e-cigarettes for cessation with or without nicotine were combined (i.e., yes to at least one) for ever tried, tried in the past year and perception that it helped (yes/no).

Sociodemographic data included age, sex, mother university-educated (yes/no) [36, 37], language spoken at home (French, English, other) and born in Canada (yes/no).

### Data analysis

Descriptive statistics were used to address the study objectives. Analyses were conducted using SPSS, Version 25.0 (IBM Corp. Released 2012. IBM SPSS Statistics for Windows, Version 25.0. Armonk, NY: IBM Corp.).

## Results

E-cigarette data were available for 775 participants (60% of 1294 participants at inception). Compared to 519 participants not retained (lost-to-follow-up and those who did not provide data in 2017–20), those retained for analysis were younger at NDIT inception in 1999 and higher proportions were female, Canadian and had university-educated mothers. Lower proportions had smoked cigarettes or other tobacco products in the past 3 months. There was no difference in language (Table 1).

**Table 1:** Baseline characteristics^a^ of participants retained and not retained (*n* = 1294). NDIT Study 1999–2020

	Retained		*p*-value^b^ for difference
	Yes (*n* = 775)	No^c^ (*n* = 519)	
Age, mean (sd)	12.7 (0.5)	12.9 (0.7)	**≤0.001**
Male, %	44.0	54.4	**≤0.001**
Mother university-educated, %	42.3	20.6	**≤0.001**
French-speaking, %	30.2	29.9	0.917
Born in Canada, %	93.8	89.6	**0.006**
Ever smoked, %	27.9	38.2	**≤0.001**
Used other tobacco products, %	8.6	12.2	**0.041**

^**a**^Measured in cycle 1 (grade 7) in 1999

^**b**^Based on t-tests and chi-square tests. Boldface indicates statistical significance (*p* < 0.05)

^**c**^Includes participants lost-to-follow-up (i.e., participants who dropped out of NDIT) and those who did not provide data in 2017–20 (i.e., participants who did not provide data in 2017–20, but did not drop out of NDIT)

One-fifth (19.2%) of the 775 participants (*n* = 149) reported past-year e-cigarette use, including more males than females (24.9% vs 14.7%; *p* **≤** 0.001). Among e-cigarette users, 50.3% used e-cigarettes less than once a month; 21.5% vaped 1–3 times per month; 16.8% vaped 1–6 times per week, and 11.4% vaped every day.

Two-thirds (63.8%) of e-cigarette users reported using only one of the three types of e-liquid investigated herein (with nicotine, without nicotine, with cannabis) in the past year, 27.5% used two types of e-liquid, and 8.7% had used all three types (Table 2). Overall, 55.0% of e-cigarette users had used cannabis-containing e-liquid (31.5% vaped cannabis e-liquid exclusively); 50.4% used nicotine-containing e-liquid (23.5% vaped nicotine e-liquid only); and 39.9% used e-liquid without nicotine (8.7% vaped e-liquid without nicotine exclusively).

**Table 2:** E-liquid used in the past-year by young adult e-cigarette users. NDIT Study 2017–20

E-liquid	n	Total^a^ %
Total	149	100.0
*One e-liquid*	95	63.8
With nicotine	35	23.5
Without nicotine	13	8.7
For cannabis	47	31.5
*Two e-liquids*	41	27.5
With and without nicotine	19	12.8
With nicotine and for cannabis	8	5.4
Without nicotine and for cannabis	14	9.4
*Three e-liquids*	13	8.7

^a^Columns may not add to 100% because of rounding

Most e-cigarette users (82.6%) had used at least one other nicotine-containing substance (i.e., conventional cigarettes, other tobacco products, cannabis mixed with tobacco) in the past year (Table 3). The mean number of other nicotine-containing substances was highest among participants who vaped nicotine-containing e-liquid. The most frequently used other nicotine-containing substance was conventional cigarettes – 72.5% of all e-cigarette users had smoked cigarettes in the past year. This proportion was highest among young adults who vaped e-liquid with nicotine, ranging from 84.6% (among those who used three e-liquid products) to 100% (among those who vaped e-liquid with nicotine or cannabis) (Table 3).

**Table 3:** Use of other nicotine-containing substances ^a^ and ND according to e-liquid. NDIT Study 2017–20

E-liquid	n	Used ≥1 other nicotine-containing substance^a^ in past year %	No. other nicotine-containing substances in past year^a^ mean (sd)	Used conventional cigarettes in past year %	Perceived nicotine dependence %	Craving %	Withdrawal symptoms^b^ %
Total	149	82.6	1.6 (1.0)	72.5	60.8	57.8	58.1
*One e-liquid*	95	75.8	1.3 (1.0)	64.2	54.3	52.7	56.6
With nicotine only	35	91.4	1.5 (0.9)	88.6	79.4	82.4	74.3
Without nicotine only	13	76.9	1.2 (1.0)	53.8	30.8	25.0	25.0
For cannabis only	47	63.8	1.3 (1.1)	48.9	42.6	38.3	47.5
*Two e-liquids*	41	95.1	1.9 (0.8)	87.8	68.3	65.9	60.0
With and without nicotine	19	100.0	1.9 (0.7)	94.7	84.2	78.9	84.2
With nicotine and for cannabis	8	100.0	2.0 (0.9)	100.0	87.5	87.5	62.5
Without nicotine and for cannabis	14	85.7	1.7 (1.0)	71.4	35.7	35.7	23.1
*Three e-liquids*	13	92.3	2.2 (0.9)	84.6	84.6	69.2	61.5

^**a**^Includes conventional cigarettes, other tobacco products (cigarillos, cigars, pipes, bidis, chewing tobacco, snuff, waterpipe) and cannabis mixed with tobacco

^b^11 participants who had never smoked cigarettes (verified in data collection cycles 1–22) were excluded

Two-thirds (60.8%) of participants perceived that they were nicotine dependent, 57.8% reported craving and 58.1% had withdrawal symptoms. Compared to participants who vaped e-liquid without nicotine or with cannabis, much higher proportions who vaped e-liquid with nicotine reported ND symptoms (Table 3).

Less than half (44.2%) of e-cigarette users had tried to quit conventional cigarettes using e-cigarettes at least once lifetime (Table 4). Of those who had tried, 80.3% were current smokers (i.e., reported smoking in the past month) at age 30.5. In the past year, 29.0% had used e-cigarettes to help them quit, 16.7% found them helpful, but 80.0% were still smoking (i.e., were current smokers). Of those who perceived that e-cigarettes were helpful as a cessation aid, 65.2% were still smoking (i.e., were current smokers). Compared to participants who vaped e-liquid without nicotine or with cannabis, higher proportions of those who used e-liquid with nicotine had attempted to quit, and reported that they were helpful.

**Table 4:** Use of e-cigarettes as a cessation aid according to e-liquid used. NDIT Study 2017–20

E-liquid used	n	Used e-cigarettes as a cessation aid		
		Ever	In past year	Perceived that e-cigarettes helped
		%	%	%
*Total*	138^**a**^	44.2	29.0	16.7
*One e-liquid*	85	40.0	25.9	14.1
With nicotine	35	77.1	54.3	28.6
Without nicotine	8	37.5	25.0	25.0
For cannabis	42	9.5	2.4	0.0
*Two e-liquids*	40	47.5	32.5	22.5
With and without nicotine	19	63.2	47.4	26.3
With nicotine and for cannabis	8	62.5	37.5	37.5
Without nicotine and for cannabis	13	15.4	7.7	7.7
*Three e-liquids*	13	61.5	38.5	15.4

^**a**^11 participants who had never smoked cigarettes (verified with past surveys) were excluded from this table

## Discussion

Mirroring the prevalence in Canada and consistent with extant studies [1, 4, 38], 19% of young adults in this study had used e-cigarettes in the past year, including relatively more males than females. Also concordant with previous reports [1, 4, 24, 25], most e-cigarette users smoked conventional cigarettes and two-thirds reported ND symptoms, with the proportion increasing to 80% among those who vaped nicotine-containing e-liquid exclusively. Only one-quarter of cigarette users had used e-cigarettes to quit in the past year. Given the popularity of vaping cannabis [39, 40] and recent vaping-induced respiratory injuries [27–29], it is critical to better understand what e-cigarette users vape and the reasons that young adults choose these products. We speculate that the reasons differ by e-liquid used.

Reflecting rapid adaptation of the market to emerging tobacco-related products, cannabis-containing e-liquid was the most frequently reported product used in e-cigarettes in this current study. The 32% of exclusive cannabis vapers (among all e-cigarettes users) in our study was markedly higher than the 7% reported by Kenne et al. (2017) [8] among US college students in 2017. The apparent increasing popularity of vaping cannabis may relate to vaporizers facilitating discrete use of cannabis - the device can be easily concealed, it has the appearance of regular e-cigarettes, and it produces near odorless vapor [10, 11, 41]. Vape from cannabis is described as more palatable [11] and because vaporized cannabis products can deliver higher concentrations of THC than conventional cannabis, it is associated with a stronger “high” [11]. Finally, users may believe that vaping cannabis is less harmful than traditional ways of consuming cannabis. Half (49%) of those who vaped cannabis exclusively in our study, smoked conventional cigarettes and 43% reported ND symptoms. Only 2% of exclusive cannabis vapers had tried to quit conventional cigarettes in the past year.

E-liquid with nicotine was the second most frequently vaped e-liquid. Half of those who used e-liquid with nicotine exclusively (i.e., 54.3% of 35) had used e-cigarettes as a cessation aid in the past year, but 60% of the 10 participant who found them helpful were current smokers. Most participants (79–88%) who used nicotine-containing e-liquid reported ND symptoms, similar to the 89% of long-term vapers reported by Etter et al. (2019) [42]. The new generation of e-liquid often contain nicotine salts which permits easier inhalation of higher levels of nicotine without the harsh taste [43], and nicotine concentrations in e-liquid have increased in the last decade [44]. While the European Union imposed a nicotine cap of 20 mg/ml, Canada has a limit of 66 mg/mL [45] and there is currently no limit in the US [46, 47]. Based on our data, users of nicotine-containing e-liquid may be seeking nicotine in a variety of nicotine-containing substances, possibly to assuage ND symptoms or for the mood-altering effects of nicotine. In using nicotine-containing e-liquid, these individuals may rely on e-cigarettes to supplement other sources of nicotine.

Finally, half of young adults who used e-cigarettes without nicotine exclusively reported smoking conventional cigarettes and 31% reported ND symptoms. However only 15% had used e-cigarettes to quit in the past year. Users of e-cigarettes without nicotine may believe as many do [4, 48], that e-cigarettes are safer and “healthier” than conventional cigarettes, they may use e-cigarettes for psychosocial reasons and/or they may be attracted to the more than 7000 flavors [7, 49]. Although many believe that the concentration of toxic chemicals released in e-cigarette vapor is lower than in regular cigarette smoke [50, 51], e-cigarette vapor contains highly oxidizing free-based nicotine, a form of nicotine that is considered highly addictive because it is easily absorbed [21, 52]. Further these products are often mislabelled so that users believe they are using nicotine-free products which actually contain nicotine [53]. Finally even nicotine-free e-cigarettes may be harmful [4] since the aerosols in e-cigarettes without nicotine have been associated with acute endothelial cell dysfunction [54].

Most NDIT participants (80%) who used e-cigarettes to help with cessation in the past year reported smoking in the past month, suggestive that e-cigarette use may not have helped many quit. Extant evidence on the efficacy of e-cigarettes is equivocal [4, 13, 14, 24, 55]. A US report using nationally representative data found that only 13% of young adult lifetime e-cigarette users reported using e-cigarettes as a cessation aid and these individuals were less likely to report cessation in the past-year [56]. It is possible that, because of nicotine in e-cigarettes (even in e-cigarettes labelled nicotine-free [53]), young adults who try to quit using nicotine-containing e-cigarettes in fact contribute to or increase their ND level [25] leaving them craving nicotine. Polytobacco users in general, are more likely to report ND symptoms than cigarette-only smokers [25, 57]. One hypothesis posits that e-cigarette users could decrease the number of cigarettes smoked because e-cigarettes deliver nicotine at similar rates, making them as or more satisfying than traditional nicotine replacement therapies, but contributing more to ND and sustained smoking [25]. Olfson et al. (2019) [56] reported that e-cigarette users were 2.3 times more likely to be daily cigarette smokers than non-e-cigarette users, and 3.6 times more likely to report a tobacco use disorder. Recent longitudinal findings concur that dual use (cigarettes and e-cigarettes) was associated with an increase in e-cigarette frequency, nicotine exposure and ND in adolescent e-cigarette users with minimal cessation benefits [58]. In addition to the nicotine content of e-cigarettes, the “nicotine environment” of e-cigarette users likely represents an important barrier to cessation [37] and contributes to continued smoking and ND.

### Future directions

The rising popularity of e-cigarettes among youth [1, 4, 56, 59] and increased availability of e-liquid with nicotine [2, 60], threatens tobacco control achievements [5]. Up to 2018, there were no regulations governing vaping products in Canada. In May 2018, the Tobacco and Vaping Products Act (TVPA) [2, 60] created a legal framework for regulating the sale, manufacture, labeling and promotion of vaping products sold in Canada including legalizing the sale of e-liquid containing nicotine. While e-cigarettes may help some smokers quit, the CDC does not recommend them for youth or people not currently using tobacco products [29]. Therefore monitoring trends in who uses which e-liquid types and the reasons for that choice is critical in terms of understanding the e-cigarette market and developing programs and policy that minimize negative impacts on health. Given changes in legislation and shifts from the original intent of e-cigarettes for cessation to their popularity for vaping cannabis, and with emerging vaping-related illness, program and policy makers and practitioners must remain abreast (or better yet, forecast) this rapidly evolving landscape to adequately prepare for the impact on health. Finally, practitioners must include vaping in their smoking cessation counselling [61].

### Limitations

Limitations of this analysis include that the proportions estimated are imprecise because the sample of e-cigarette users was small. Results may not be generalizable, although the proportion of e-cigarette users in NDIT mirrored national data, as did the observation that more males than females used e-cigarettes [1]. E-liquid content with respect to nicotine content was “perceived.” Even if information on nicotine was available and participants remembered accurately, the nicotine content of e-liquid is often mislabeled [53]. Data for this study were collected pre- and post-TVPA, which could have increased access to e-cigarettes with nicotine. However, e-liquid with nicotine were widely available online and in stores prior to the TVPA [2, 62]. Improved product labelling (i.e., including the concentration of nicotine, complete listings of ingredients on packages, presence of e-cigarette health warnings) following the TVPA could have impacted product choice. Results from a recent study [63] suggest that 28% of e-cigarette users purchase their e-liquid online where regulations are not always adhered to [62, 63]. Evidence from Australia which banned the sale of nicotine vaping products, suggests that regulation is associated with increases in online sales of vaping products [63]. ND indicators measured ND pertaining to conventional cigarettes. Finally the study design was cross-sectional limiting causal inference.

## Conclusions

A popular use of e-cigarettes in this 2017–20 sample of young adults was to vape cannabis. Relatively few used e-cigarettes for cessation and most used other nicotine-containing substances including combustible cigarettes. Identification of emerging trends in e-cigarette use is needed to inform programs and policy.

## Data Availability

Data are available upon request. To gain access, applicants must complete a data access form available on our Nicotine Dependence in Teens website (www.nditstudy.ca) and return it to the principal investigator (jennifer.oloughlin@umontreal.ca). The procedure to obtain access to Nicotine Dependence in Teens data is described in O’Loughlin, J., Dugas, E. N., Brunet, J., DiFranza, J., Engert, J. C., Gervais, A., Gray-Donald, K., Karp, I., Low, N. C., Sabiston, C., Sylvestre, M. P., Tyndale, R. F., Auger, N., Belanger, M., Barnett, T., Chaiton, M., Chenoweth, M. J., Constantin, E., Contreras, G., Kakinami, L., Labbe, A., Maximova, K., McMillan, E., O’Loughlin, E. K., Pabayo, R., Roy-Gagnon, M. H., Tremblay, M., Wellman, R. J., Hulst, A., Paradis, G., 2015. Cohort Profile: The Nicotine Dependence in Teens (NDIT) Study. Int J Epidemiol. 44(5), 1537–1546. doi: 10.1093/ije/dyu135. The relevant paragraph is described below. This process has been approved by the ethics committee at the CRCHUM. Access to Nicotine Dependence in Teens data is open to any university-appointed or affiliated investigator upon successful completion of the application process. Masters, doctoral and postdoctoral students may apply through their primary supervisor. For more information, visit www.nditstudy.ca or contact the Principal Investigator.

## Abbreviations

CDC: Centers for Disease Control and Prevention;
ND: Nicotine dependence;
NDIT Study: Nicotine Dependence in Teens Study;
SD: Standard deviation;
THC: Tetrahydrocannabinol
TVPA: Tobacco and Vaping Products Act
